# LSDDEP2: study protocol for a randomised, double-dummy, triple-blind, active placebo-controlled, parallel groups trial of LSD microdosing in patients with major depressive disorder

**DOI:** 10.1186/s13063-024-08384-3

**Published:** 2024-08-24

**Authors:** Dimitri Daldegan-Bueno, Carina Joy Donegan, Anna Forsyth, Rachael Louise Sumner, Robin J. Murphy, David B. Menkes, William Evans, Nicholas Hoeh, Frederick Sundram, Lisa M. Reynolds, Rhys Ponton, Alana Cavadino, Todd Smith, Partha Roop, Nathan Allen, Binu Abeysinghe, Darren Svirskis, Mahima Bansal, Suresh Muthukumaraswamy

**Affiliations:** 1 School of Pharmacy, Faculty of Medical and Health Sciences, University of Auckland, 85 Park Road, Grafton, Auckland, 1023 New Zealand; 2 Department of Psychological Medicine, Faculty of Medical and Health Sciences, University of Auckland, 22 Park Avenue, Grafton, Auckland, 1023 New Zealand; 3 Department of Psychological Medicine, Waikato Clinical Campus, University of Auckland, Pembroke Street, Hamilton, 3240 New Zealand; 4 Mana Health, 7 Ruskin St, Parnell, Auckland, 1052 New Zealand; 5 School of Population Health, Faculty of Medical and Health Sciences, University of Auckland, 22 Park Avenue, Grafton, Auckland, 1023 New Zealand; 6 Te Whatu Ora, Auckland, 1023 New Zealand; 7 Faculty of Engineering, University of Auckland, Auckland, 1023 New Zealand

**Keywords:** Microdosing, Psychedelics, LSD, Major depressive disorder, Randomised controlled trial

## Abstract

**Background:**

Major depressive disorder (MDD) poses a significant global health burden with available treatments limited by inconsistent efficacy and notable side effects. Classic psychedelics, including lysergic acid diethylamide (LSD), have garnered attention for their potential in treating psychiatric disorders. Microdosing, the repeated consumption of sub-hallucinogenic doses of psychedelics, has emerged as a self-treatment approach for depression within lay communities. Building upon preliminary evidence and the successful completion of an open-label pilot trial of microdosing LSD for depression (LSDDEP1), this protocol outlines a phase 2b randomised controlled trial (LSDDEP2). The main objective of LSDDEP2 is to assess the modification of depressive symptoms, measured by the Montgomery–Åsberg Depression Rating Scale (MADRS), following a regimen of LSD microdoses versus placebo.

**Methods:**

This is a randomised, double-dummy, triple-blind, active placebo-controlled, parallel groups trial of LSD microdosing in patients meeting DSM-5 criteria for major depressive disorder. Participants will undergo an 8-week LSD microdosing regimen using the titratable MB-22001 formulation taking two doses a week. All doses will be self-administered at home and will be titratable from 4 to 20 μg based on subjective perception and tolerability. In addition to depression symptoms, outcome will include psychiatric and personality inventories, sleep and activity tracking, electroencephalography (EEG), blood biomarkers, semi-structured interviews, and safety (e.g. adverse event, laboratory exam) measures.

**Discussion:**

This study will be the first randomised controlled trial to administer controlled microdoses of LSD for treatment of MDD in participants’ naturalistic environment. The measures included are designed to assess the drug’s safety, mechanism, and treatment efficacy over placebo in this population. The results of this study will be important for assessing the viability of psychedelic microdosing as an additional treatment option and for informing the direction of future clinical trials.

**Trial registration:**

ANZCTR, ACTRN12624000128594. Prospectively Registered on 13 February 2024.

**Supplementary Information:**

The online version contains supplementary material available at 10.1186/s13063-024-08384-3.

## Background

Worldwide, major depressive disorder (MDD) is the leading cause of disease burden in terms of mental health, affecting approximately 5% of the global population (or approximately 280 million people) [1, 2]. Available treatments lack efficacy in approximately one third of patients and often have considerable side effects [3–5]. In the context of high prevalence and limited treatment options, new, effective interventions are essential to reduce the impact of MDD. Classic psychedelics, substances known to produce profound alterations in consciousness and perception linked to the activation of 5-HT2A receptors [6], are increasingly being investigated for their therapeutic potential in psychiatric disorders [7]. The phenomenon of microdosing psychedelics, i.e. the repeated consumption of doses lower than the threshold to cause substantial alterations in consciousness [8], has emerged strongly in the last decade among communities of people who use drugs [9]. Although the reasons for microdosing psychedelics vary, microdosing as an attempt to self-treat or alleviate depressive symptoms appears to be one of the most frequent motivators [10, 11]. Scientific evidence indicates that microdoses (5–20 μg) of lysergic acid diethylamide (LSD) can acutely affect neural connectivity, cognition, and mood in healthy volunteers, with no serious adverse events reported among eight controlled trials [12]. An intriguing question is the extent to which the mood-elevation caused by microdosing LSD observed in healthy volunteers [13] may have therapeutic value for people suffering from depression.

In this study, we therefore seek to determine whether a regimen of LSD microdoses delivered with a mobile-phone-based psychological intervention can modify depressive symptomatology in patients with MDD relative to active placebo (caffeine or methylphenidate). Towards this goal, a phase 2a, open-label pilot trial (LSDDEP1) was conducted to determine the tolerability of LSD microdoses in patients with MDD and to assess the feasibility of proceeding with a phase 2b randomised trial (LSDDEP2) [14]. Although not yet published, results from LSDDEP1 (*N* = 19) indicate that the regimen was well tolerated, with no serious adverse events reported in a sample of people with MDD. Compliance with the trial assessment load was high, indicating that conducting the phase 2b controlled trial is feasible. On this basis, the current work registers the protocol for a phase 2b placebo-controlled trial of microdosing LSD for MDD (LSDDEP2).

## Objectives

The primary objective of LSDDEP2 is to determine whether microdosing LSD can modify symptoms of MDD as measured by the Montgomery-Åsberg Depression Rating Scale (MADRS) global score. Our secondary objectives are to determine whether microdosing LSD can modify symptoms of anxiety, stress, rumination, and anhedonia as well as quality of life in patients with MDD using domain specific scales. Additionally, we seek to describe the safety profile of microdosing LSD in patients with MDD using self-reported adverse event and laboratory measurements. Trial registration: ACTRN12624000128594. Registered 13 February 2024, https://www.anzctr.org.au/Trial/Registration/TrialReview.aspx?id=387101&isReview=tru>.

## Study design

LSDDEP2 is a phase 2b randomised, double-dummy, placebo-controlled parallel-groups trial designed to determine superiority of LSD versus placebo in a two-arm design. LSDDEP2 will be triple-blinded, with participants, investigators, and outcome assessors blinded to the intervention. Eligible participants (*N* = 90) will receive LSD microdoses (titrated from 4 to 20 μg) or an active placebo (caffeine or methylphenidate). The main allocation ratio to LSD placebo is 1:1 and within the placebo group the caffeine to methylphenidate allocation ratio is also 1:1. Participants will self-administer all but one of the doses over an 8-week period, taking two doses a week on non-consecutive days. This protocol followed SPIRIT reporting guideline [15] and the checklist can be accessed as the Additional file 1.

### Study population

To participate in the trial, participants must meet all the inclusion criteria and none of the exclusion criteria and adhere to the lifestyle considerations which are outlined in Table 1. Screen failures are defined as participants who consent to the clinical trial but are not subsequently entered into the study. Participants can take part in the trial whilst undergoing antidepressant therapy. However, participants will be asked to not start any new treatment for depression once enrolled in the trial. Screen failures will only be computed for those who signed the informed consent and, aiming to meet the Consolidated Standards of Reporting Trials (CONSORT) publishing requirements, we will collect the following minimal set of screen failure information: demography, screen failure details, eligibility criteria, and any serious adverse event. A minimum of 25% Māori or Pasifika participants will be recruited.

**Table 1:** List of inclusion and exclusion criteria and life lifestyle considerations for LSDDEP2

**Inclusion criteria**	
Consent	-Provision of signed and dated informed consent form -Stated willingness to comply with all study procedures and availability for the duration of the study -For heterosexually active persons of child-bearing potential: agree to use an effective or highly effective contraception for at least 1 month prior to screening and agreement to use such a method until the 1-month follow-up is completed -For heterosexually active males of reproductive potential: use of condoms or other methods to ensure effective contraception with a partner -Ability to take oral medication and be willing to adhere to the study intervention regimen -Agreement to adhere to lifestyle considerations throughout the study duration
Demographics	-Any gender identity -Aged, 21–65 years
Clinical characteristics	-Diagnosis of MDD as per the DSM-5 criteria for MDD (determined by clinical interview) -Have a MADRS score between 18 and 35 at the time of screening
**Exclusion criteria**	
Mental health diagnosis	-Current or past history of schizophrenia or other psychotic disorders or bipolar I or II disorder as assessed by clinical interview. Patients with MDD with psychotic features will be excluded. Also excluded will be individuals with a known first-degree relative with these disorders -Diagnosis of PTSD -Diagnosis of an eating disorder
Current risk	- Risk of suicide as determined by the Columbia-Suicide Severity Rating Scale (C-SSRS). Specifically, patients answering “yes” to items 3–5 covering the last 3-month period will be excluded -Stage II or higher treatment-resistant depression as defined by the Thase and Rush [30] staging criteria for the current depressive episode
Drug use	-Substance dependence in the previous 6 months as assessed by clinical interview with a New Zealand modified version of the NM-ASSIST -Problematic use of alcohol defined as a score on the AUDIT of 16 or greater -Any lifetime history of psychedelic microdosing -Use of serotonergic psychedelic drugs (LSD, psilocybin, DMT, etc.) in the last 3 months -Lifetime history of self-medicating with psychedelics to treat their depression -Excessive sensitivity to caffeine -Daily use of caffeine > 500 mg
Physical health	-BMI < 18 and > 35 -Planned or current pregnancy or lactation
Vital signs	-Cardiovascular conditions including abnormal heart rate or blood pressure to be checked at screening. A threshold of exceeding 160 mmHg (systolic) and 90 mmHg (diastolic), averaged across three assessments taken on the screening day will be used. Participants with well-managed hypertension will not be excluded
Laboratory tests	-Significant renal or hepatic impairment -Abnormal 12-lead ECG as judged by a study physician -Abnormal laboratory test findings (complete blood count, liver function test, renal function test, thyroid function test) as judged by a study physician
Diagnoses	-Any unstable medical or neurological condition -Any other condition judged by the treating clinician as likely to impact on the ability of the participant to complete the trial
**Lifestyle considerations**	
Caffeine	Limit caffeine consumption to ~ 100 mg on dosing day
Alcohol	Abstain from alcohol for 24 h before the start of each first dosing session. A breathalyser test will be performed at each dosing session. A failed breath alcohol test (> 0) will lead to withdrawal
Recreational drugs	Abstain from recreational drugs for the duration of the study. A urine drug screen will be taken at screening and baseline. A failed drug test at baseline will lead to withdrawal
Tobacco	Participants who use tobacco products will be instructed that use of nicotine-containing products (including nicotine patches) will not be permitted whilst they are at the study site
Depression therapy	Not begin any new therapies for depression over the course of the study. This would lead to withdrawal from the study
Menstruation	For persons who are menstruating, best efforts will be made to time the baseline, dosing, and measure sessions with the start of the follicular phase of the menstrual cycle. Participants will be asked to report the onset of menses during their participation

### Study intervention and supply

#### Investigational Medicinal Product (IMP) (MB-22001), inactive placebo and double-dummy placebos

MB-22001 will be manufactured by Biocell Corp NZ Ltd under a GMP Manufacturing licence issued by MedSafe New Zealand using LSD hemi-tartrate API (Psygen Ltd, Canada). Each vial of MB-22001 contains 20 μg of LSD free-base equivalent in an ethanol–water mixture. Inactive matched placebo vials are identical in all respects, with no LSD API present. LSD is colourless, flavourless, and odourless. The double-dummy placebo product will be 50 mg caffeine or 10 mg methylphenidate capsules compounded by CompoundLabs NZ with inactive double-dummy placebo capsules being identical in appearance.

Participants will be supplied eight doses at the baseline visit which will be taken at home. Participants will then return to the clinic to take their nineth dose at the dosing visit and then will leave with a supply of a further seven doses (doses 10–16). Following completion of the dosing protocol, participants will return to the clinic for a measure visit. To ensure safety and prevent accidental ingestion by minors, all participants will be offered a lock box to securely store the IMP/placebo at home. Participants will be asked to dispose of the packaging and residual dose and to bring back the placebo/matched placebo capsules to allow for capsule counting.

#### Dosing and administration and titration

The majority of drug interventions (15/16) will be self-administered out of the lab (e.g. at home). For each self-administration, participants will take the appropriate amount of MB-22001 sublingually. Then, they will swallow the active or matched placebo capsules whole. Participants are instructed to take doses before 2 pm each day to prevent disruption to sleep and not to drive or engage in dangerous activities for a 6-h window following dosing.

With the aim of reducing the likelihood of negative side effects and maximise the therapeutic potential, we will use a titration protocol in which the participants will determine dose increments based on their subjective experience of drug effects, similar to our previous MDLSD and LSDEP1 studies [13, 14]. On each dosing day, participants will complete a five-point Likert scale indicating whether they thought the dose was too much, too little or adequate. They will be informed that if they experience any disturbance of daily functioning, they should decrease the dose for the next dosing. The starting dose of 8 μg will be increased or decreased by 2 or 1 μg increments at each dosing to a maximum and a minimum of 20 μg and 4 μg, respectively. The double-dummy placebo or matched placebo capsules will also be titrated accordingly. The initial dose of caffeine will be 100 mg (2 capsules) and can be increased to a maximum of 300 mg (6 capsules) and decreased to 50 mg (1 capsule). The initial dose of methylphenidate will be 20 mg (2 capsules) and can be increased to a maximum of 60 mg (6 capsules) and decreased to 10 mg (1 capsule).

### Accompanying psychotherapeutic intervention

The drug intervention will be accompanied by a psychotherapeutic intervention aiming to maximise the potential psychological effects of microdosing by setting intentions for their experience. For each microdosing session, participants will be encouraged to engage in a self-selected psychologically beneficial activity. This is based on results of the MDLSD study, in which qualitative reports indicated that participants generally had more positive experiences when microdosing coincided with psychologically beneficial activities. At the baseline session, an initial set of activities will be selected with assistance from a member of the trial team. However, participants will be able to change, add or remove activities as they wish.

### Study mobile phone app and Mobile Directly Observed Therapy (MDOT)

A custom study application will be installed on each participant’s mobile phone to help with compliance and intervention. The mobile app will guide participants through each double dummy administration using Mobile Directly Observed Therapy (MDOT), which ensures adherence and prevents medication stacking. The MDOT was used successfully in previous trials [13, 14, 16]. On dosing days, participants will receive a notification via the mobile app reminding them to take their medication in the morning and will be asked to record a video of themselves doing so. Trial staff will review the video to ensure compliance with instructions. Videos will be deleted after compliance checking and documented in the electronic case report form (eCRF). Participants will receive training on the MDOT procedure during the Baseline session. Also recorded will be engagement with the mobile app and compliance with the therapeutic regimen, including the number of doses where the intention was set, and journaling conducted. If a participant consistently performs the MDOT procedure poorly, they may be removed from the trial at the discretion of the Principal Investigator.

### Study sessions

The trial consists of three on-site visits and five online video sessions (two on trial, and three follow-ups) plus two screening sessions (one online and one on-site). All on-site visits will occur at the Clinical Research Centre at the University of Auckland Grafton Campus in Auckland, New Zealand. A list of sessions and scheduled activities based on the SPIRIT checklist [15] is presented in Table 2.

**Table 2:**
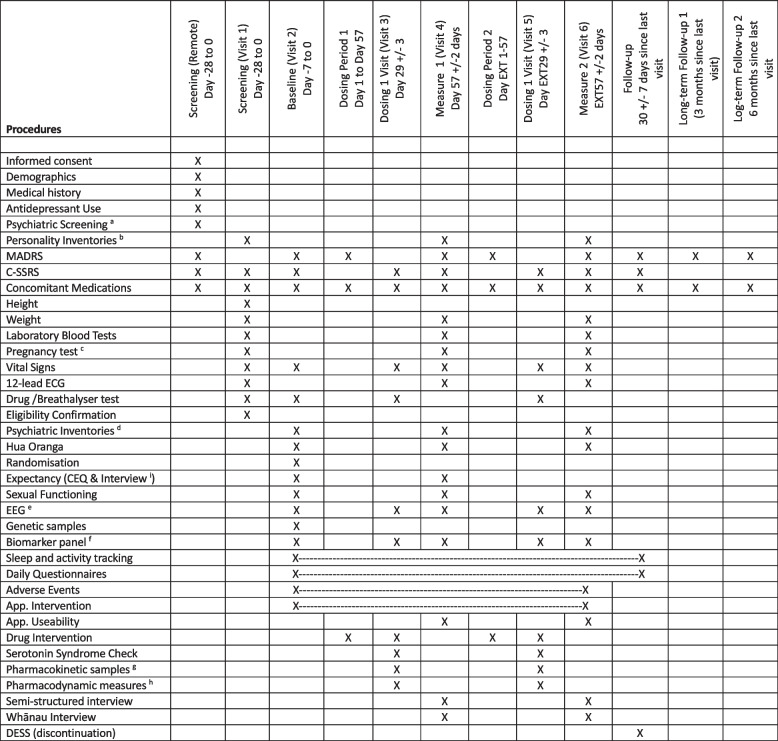
Schedule of activities and measures of LSDDEP2

^a^AUDIT, NM-ASSIST and Clinical Interview

^b^bBFI-2, CFI, MODTAS, FFMQ

^c^cSerum pregnancy test (persons of childbearing potential).

^d^HAM-A, RRS, DARS, DASS-21, WHOQOL-BREF, WCS

^e^Resting EEG eyes-open, eyes-closed, long-term potentiation, mismatch negativity, Doors task, LDAEP

^f^Plasma BDNF, mRNA, inflammatory cytokines, SNPs.

^g^PK samples at baseline and at 0, 20, 40, 60, 90, 120, 180, 240, 360 (+/-5 minutes) minutes after drug administration. The actual time will be recorded.

^h^Vital signs, VAS measures, speech task measured at each PK sample-point

^i^Expectancy Interview only conducted at Baseline. Conducted at measure only if entering the extension period.

#### Pre-screening

Upon expressing interest in the trial, participants will receive an email containing a link to a brief pre-screening questionnaire. After completing the pre-screening questionnaire, eligible participants will receive an email containing the relevant participant information sheet (PIS) and consent form (see Additional File 2). These documents contain details about the trial objectives, participant responsibilities, known risks, and any protocol implications and constraints. The PIS will be provided to potential participants prior to their screening session, affording them ample time to seek independent advice. Then, a research team member will discuss the main trial requirements and exclusion/inclusion criteria via telephone. If the participant remains eligible and interested in the trial, they will be invited to screening.

#### Screening (days − 28 to 0)

The screening process will be divided into two sessions. The first session will be remote via video call. Informed consent will first be obtained by a trained research team member through verbal confirmation of understanding the PIS and written consent (digital signature on the consent form). In this call, medical and psychiatric history and current psychiatric status will be assessed. If deemed eligible, participants will be invited to an in-person screening session, which includes physical measurements such as height, weight, vital signs, a 12-lead ECG, laboratory blood tests, a drug/alcohol breathalyser test, and, when applicable, a pregnancy test.

#### Baseline visit (days − 7 to 0)

The baseline visit is the first trial session and will include EEG recordings, assessment of depression and expectancy, administration of psychiatric inventories, and collection of blood samples for biomarker analysis (see Table 2 for a more detailed outline of these assessments). Participants will receive a wearable activity tracker, and the study app will be installed on their mobile phones. In this session, recording possible adverse events, tracking sleep and activity, and completing daily questionnaires will commence. Additionally, participants will be trained on how to use the phone app and administer their doses at home. At the conclusion of the session, participants will be given a box containing 8 doses of the IMP or inactive placebo and matched double-dummy placebo capsules.

#### Dosing period (days 1 to 57)

Participants will begin their home dosing regimen one to seven days following the baseline session. The IMP or inactive placebo will be self-administered and will be supervised through MDOT as described previously. During this period, participants will undergo MADRS assessments 2, 4, and 6 weeks after the Baseline visit via video call.

### *Dosing visit (days 30* ± *3)*

Participants will receive their ninth dose during an in-clinic visit. Blood samples and subjective drug effect measures will be collected before drug administration and at intervals of 20, 40, 60, 90, 120, 180, 240, and 360 min (with a variance of ± 5 min) after IMP administration. EEG measures will commence approximately 2 h after administration. Additionally, participants will undergo the 4-week MADRS assessment before dosing. For this visit, we will use their individually titrated dose, which can be considered their “therapeutic” dose. If scheduling constraints prevent the dosing day from occurring precisely on the ninth dose, it will be conducted as closely in dose sequence as possible. Participants will leave the dosing visit with an additional seven doses (doses 10–16).

#### Measure visit (day 57 ± 2)

This will be the last on-site visit. Participants will undergo the 8-week MADRS assessment, EEG, blood biomarker analysis, and semi-structured interview. If they agree, a family member will also be invited to participate in a separate semi-structured interview to discuss their whānau/family members’ perception and experience of the participant taking part in the trial.

#### Extension

All participants will have the option to enter an 8-week open-label extension period following the same protocol as the initial phase of the trial, including the dosing period, dosing visit, and measure visit. The start of the extension period can be delayed by up to 14 days from the measure visit, and participants may choose to dose less than twice a week without it being considered a protocol violation. The measure session for extension will be done after 8 weeks of the commencement of the extension, regardless of the quantity of doses the participants took. If a participant discontinues microdosing midway through the extension period, they will be invited for a final measurement session and then enter the follow-up period.

#### Follow-up

Participants will undergo three follow-up assessments at 1, 3, and 6 months (± 7 days) from the last measure visit. MADRS will be assessed in all follow-up sessions, and Discontinuation Emergent Signs and Symptoms (DESS) will be measured in the first session.

## Study assessments and procedures

### Outcomes

The main efficacy measure for LSDDEP2 will be the MADRS, an interview-based depression scale commonly used and recommended in pharmaceutical/regulatory registration trials of depression [17–19]. It consists of 10 items, which are summed to a maximum potential score of 60 [20]. Therefore, the primary efficacy outcome for LSDDEP2 is the 8 weeks MADRS, assessed during the measure session by a trained research member. Secondary measures include anxiety, stress, rumination, anhedonia, and quality of life using domain-specific scales. Exploratory measurements include personality inventories, expectancy, and EEG and blood-based biomarkers. Safety measures include recording adverse events and laboratory results. Adverse events and their severity will be recorded in the mobile app off-site and by the research team on-site and will be coded using the Medical Dictionary for Regulatory Activities (MedDRA). A comprehensive description of the primary, exploratory, and safety outcomes is provided in Tables 3 and 4.

**Table 3:** Primary and secondary outcomes for LSDDEP2

Outcome domain	Measure	Definition
**Primary outcome**		
MDD symptoms	MADRS assessed at baseline and at 2, 4, 6, and 8 weeks timepoints	10 items, each clinician-rated on a 7-point Likert scale, summed to give a total score between 0 and 60
Anxiety symptoms	HAM-A, assessed at baseline and at the 8-week timepoint	14 items, clinician rated on a 4-point Likert scale, these scores combined to give a final composite score
Depression, stress, and anxiety symptoms	DASS-21, assessed at baseline and at the 8-week timepoint	21-items, 5-point Likert scale, from 0 (never) to 4 (almost always). Three subscales, reported as summed scores
Ruminative symptoms	RRS, assessed at baseline and at the 8-week timepoint	22 items, four scales measuring two aspects of rumination, rated from 1 (almost never) to 4 (almost always)
Anhedonia symptoms	DARS, assessed at baseline and 8-week timepoint	4 domains that call for participant examples, rated on a 5-point Likert scale (0-4). Total and each of its four subscales will be compared
Quality of life	WHOQOL-BREF, assessed at baseline and 8-week timepoint	26 items, scored on a 5-point Likert scale. Each of its four subscales will be compared

**Table 4:** Exploratory and safety outcomes for LSDDEP2

Outcome domain	Measure	Definition
Exploratory outcomes		
Connectedness	WCS, assessed at baseline and 8-week timepoint	23 items marked on a visual analogue scale between 0 (“not at all”) and 100 (“entirely”). Each of the three subscales will be compared
Te Whare Tapa Whā	Hua Oranga, assessed at baseline and 8-week timepoint.	16 items where each of the constructs are scored by participants on a 5-point scale with descriptors of each provided. Each of its four subscales will be compared
Daily mood	HAMD-6 self-report will be assessed from baseline daily through until follow-up 1	6-item scale, clinician rated. Captures core features of depression
EEG	The EEG LTP, MMN, LDAEP, and doors tasks as well as resting state will be measured at baseline, dosing, and measure timepoints	Recorded with 64 channel caps, 5 min eyes open 5 min eyes closed resting state, sensory LTP with vertical and horizontal sine gratings on grey background, MMN 5–11 sinusoidal tones, classic oddball response, LDAEP measures serotonergic function, 100 Hz stimulus tones at differing intensities, doors measure reward processing (positive ERP ~ 250–300 ms after feedback)
Plasma/serum BDNF and mRNA biomarkers	Plasma/serum BDNF samples will be measured baseline. Dosing (pre and 6 h post) and measure timepoints	BDNF plasma levels, expression of mRNA markers
Genotype prediction of outcome measures	Whole blood samples will be taken from participants at baseline	Polymorphism of relevant genes
Personality trait modification	BFI-2, CFI, FFM-Q, and MODTAS are measured at screening and measure points	BFI 60 items on Likert 1–5, for main personality traits, CFI 20 items from 1 to 7 giving total score and 2 subscales, FFM-Q 38 items on 5-point Likert Scale, 34 items on a 5-point Likert scale
Expectancy	CEQ will be measured at baseline and dosing sessions. Semi-structure expectancy interview will be done at baseline	6 items scores on a 9-point Likert scale
Sleep, activity, physiology	Fitness tracker data will be recorded continuously from baseline through to one month after completion of regimen	Wearing of a Garmin Activity tracker logging basic measurements including sleep duration, quality, physical activity, heart rate, and stress levels
Daily experience	Daily VAS scales completed every evening in the study app. Participants will be encouraged to record an audio journal in the study app	HAMD-6 self-report with VAS scales for connected, creative, energy, happy, irritable and jittery. Adverse event reporting
Withdrawal/discontinuation symptoms	DESS will be measured at the follow-up 1 timepoint	Checks for symptoms of discontinuation syndrome
Acceptability of study app	Acceptability questionnaire	5 items considering useability of the app
Engagement with therapeutic journalling	Journal entries	Number of journal entries accessed via the app
Subjective experience	Semi-structured interview	Conducted with the participant (and optional whānau member) to record their subjective experiences of being involved in the trial
Plasma pharmacokinetics	Pharmacokinetic samples will be taken at baseline and 20, 40, 60, 90, 120, 180, 240, and 360 min (± 5 min) after the in-clinic dose	4 mL of blood collected
Pharmacodynamics	Pharmacodynamic measurements will be recorded at baseline 20, 40, 60, 90, 120, 180, 240, and 360 min (± 5 min) after the in-clinic dose	Vital signs, speech task, VAS scales
Extension period	An intervention extension period is offered to all patients for 8 weeks	The number of doses administered in this period is the endpoint
Durability of antidepressant response	MADRS assessments will be conducted at three follow-up time points (1, 3, and 6 months after the final measure session)	10 items, each clinician rated on a 7-point Likert scale, summed to give a total score between 0 and 60
Safety outcomes		
Adverse event profile	Assess the incidence of SAEs and AEs by severity, recording in the app and on-site	Tabulations of AEs by severity and SAE listings from baseline to 1-month post-intervention
Objective safety measures	Laboratory tests	Complete blood count, liver function, renal function, thyroid function), 12-lead ECG, vital signs will be measured at baseline and at the 8-week timepoint and after completion of extension period

### Participant recruitment

LSDDEP2 will aim to enrol 90 patients with a target at least 25% of the sample self-identifying as either Māori or Pasifika ethnicity. Some prospective participants may be excluded based on ethnicity if goals are not met. Recruitment will occur from the community, primarily through general practices in the greater Auckland (New Zealand) area and through advertisements in local newspapers, noticeboards, and online platforms such as social media. Also, our study team maintains a database of individuals with self-reported MDD who have shown interest in participating in clinical trials which will be utilised. Participants will have reimbursement for any incurred expenses and will receive a koha of $250 in gift cards upon completion of the study. Additionally, they will receive extra compensation for each completion of the doors EEG task (on average $20 per task). Participants who do not pass the screening process will receive a $20 gift card for their participation.

### Randomisation and blinding

For randomisation, a biostatistician (author AC) will generate a code list with participants randomised in blocks with a 1:1 allocation ratio with variable block sizes. The total code list length will be 90, with 45 codes for each intervention arm. To ensure allocation concealment, a blinded investigator will allocate participants at the point of randomisation to the first available code on the randomisation sequence list.

All staff except the pharmacist (author RP) and the biostatistician will be blinded. To reduce bias, study pharmacists and the biostatistician will never directly interact with trial participants. Outcome assessors for the main measure (MADRS) will also be blinded and will not interact with the participants from the time of first IMP administration except for MADRS assessments. They will only have access to forms related to depression assessment. MADRS interviews will be audio recorded and a sub-sample of recordings will be cross-scored. Outcome assessors will avoid discussing patients with trial coordinators and participants will be asked not to share drug experiences with them. All trial staff will remain blinded until the study database is locked after completion of the last month follow-up (end of trial). In the event of inadvertent blinding breaking, this will be described and recorded in the CRF for a participant as a protocol deviation. In case of emergency, the study pharmacist and biostatistician will also keep an electronic spreadsheet of allocations so that de-blinding can be performed rapidly.

### Data collection and management

Each participant will have individual paper-based files maintained, though most of the case report form and data capture will be managed through the online Research Electronic Data Capture (REDCap) tools hosted at the University of Auckland. All electronic data will be stored on password-protected University servers. Each data file will have a corresponding original, unprocessed version that can only be modified by the University Systems Administrators, ensuring audit capability and accuracy of extracted data.

Participant confidentiality and privacy are strictly held in trust by the participating investigators, their staff, collaborators, students and the study sponsor. This confidentiality is extended to cover testing of biological samples and genetic tests in addition to the clinical information relating to participants. Therefore, the study protocol, documentation, data, and all other information generated will be held in strict confidence. No information concerning the study or the data will be released to any unauthorised third party without prior written approval of the sponsor. The study participant’s contact information will be securely stored at each clinical site for internal use during and at the end of the study. All research activities will be conducted in as private a setting as possible. De-identified biological samples will be stored at the University of Auckland and used only for research related to microdosing and/or depression. During the conduct of the study, an individual participant can choose to withdraw consent to have biological specimens stored for future research.

### Sample size estimation

This is the first study to investigate LSD microdosing as an intervention for MDD; thus, prior estimates of effect size for power calculations are lacking. Given the intention to explore secondary and exploratory outcomes with optimal power, sample size was based on pragmatic reasons (cost and potential ability to recruit participants). Nevertheless, a sensitivity analysis of the primary outcome was conducted for the fixed sample size of 90 (with 45 participants per group). Monte Carlo simulations were conducted in R statistical software using the mixed effect models [21] with 10,000 simulations per run. Data were simulated on each iteration using the following parameters (*N* = 90, 5 dropouts with data missing at random, *α* = 0.05, (1-*β*) = 0.8, mean baseline MADRS score of 30, random effects distributed as *η* ~ *N*(0, 6.32), and errors distributed as *e* ~ *N*(0, 4.89). These standard deviation estimates were obtained from linear mixed-effect models that fit data from a previous ketamine antidepressant trial conducted in our laboratory [22]. Monte Carlo simulations revealed that the LSDDEP2 will be powered to detect changes as smalls as ~ 4.2 MADRS points for the difference between groups at 8 weeks.

### Data analysis

A full statistical analysis plan (SAP) will be written and completed before the end of the trial (before database lock and unblinding of investigators) and submitted to the trial data monitoring committee which will define analysis sets and the primary efficacy analysis. The primary efficacy analysis will use linear mixed effects models to determine the difference in groups (LSD versus placebo) at the 8 week MADRS measurement point quantified using the Group x Day interaction effect with the alpha threshold set at *p* = 0.05. No interim analysis of the primary outcome measure will be conducted during the trial. The SAP will be published alongside reports of the primary efficacy outcome.

### Study intervention discontinuation and participant withdrawal

The intervention will be halted immediately if a participant: requests it, violates any exclusion criteria, demonstrates inadequate dose compliance, experiences a serious adverse event, or encounters any other condition that the study team judge likely to affect their ability to function on their day to day. Withdrawal decisions will be made at the discretion of the study clinicians. Participants are free to withdraw from the study at any time upon request. The reason for participant discontinuation or withdrawal from the study will be recorded on the case report form.

Participants who sign the informed consent form, are randomised, receive the study intervention and subsequently withdraw, or are withdrawn or discontinued from the study will not be replaced. However, randomised participants who have not yet received the study intervention may be replaced—in such a case, the code will be reallocated to a subsequent participant. A participant will be considered lost to follow-up if they fail to return for one scheduled visit and cannot be contacted by the study site staff.

### Safety oversight

A subset of investigators from the study will comprise the trial steering committee (TSC), whose role is to oversee the trial comprehensively. The TSC will collectively develop and approve the final protocol, monitor the trial’s progress and adherence, ensure participant safety, consider new information, and manage publication and dissemination. The TSC reached a full consensus before the final protocol was submitted and will take responsibility for major decisions such as changing the protocol and supervising trial progress. For any necessary protocol changes, at least 50% of the investigators, including the principal investigator (PI), must agree.

Data monitoring will be overseen by an independent data monitoring committee managed by the Health Research Council of New Zealand Data Monitoring Core Committee. Meetings to review data will be held every 6 months starting from the trial’s initiation until its conclusion. The trial statistician or their representative will prepare both open and closed reports for each meeting. These reports will be submitted to the Health Research Council 14 days before each meeting. Clinical site monitoring will also be carried out to ensure the protection of trial participants’ rights and well-being, the accuracy and completeness of reported trial data, and compliance with the International Conference on Harmonisation Good Clinical Practice (ICH GCP) guidelines, approved protocol/amendment(s), and relevant regulatory requirements. Certified clinical trial monitors from the National Institute of Health Innovation (www.nihi.ac.nz) will conduct the clinical trial monitoring independently.

### Concomitant care and post-trial care

During the study, participants will receive regular care from their general practitioner and will be offered recommended therapies for any non-exclusionary health conditions that may occur. Whilst the likelihood of participants experiencing long-term harm is considered very low, participants can seek compensation for any trial-related injuries through the University of Auckland’s insurance policy.

### Role of patient and public involvement panel

The LSDDEP2 clinical trial protocol was developed in consultation with a panel of twelve patients from the Auckland community with lived experience of depression. In a series of forums, panellists provided input regarding clinical trial design and feedback on the development of patient facing documents (PIS and consent forms). Input from the panel was given on an advisory basis to the Investigators. Panellist involvement is on-going and expected to continue through to the dissemination of results from LSDDEP2.

## Dissemination policy

This study will be registered at ANZCTR, and results information from this trial will be available within 12 months of completion of the study. Every attempt will be made to publish results in peer-reviewed journals. A lay summary of results will be provided to trial participants who opt-in and will be presented to the patient and public involvement panel.

## Discussion

This is the first controlled trial to investigate the therapeutic potential of repeated self-administrated microdoses of a psychedelic drug in people with depression in a naturalistic setting.

A notable strength of this protocol is that participants undertake microdosing at home as part of their day-to-day life and engage in previously decided self-selected activities. Not only does this approach increase ecological validity, as it is closer to what is being commonly done by the lay community, but it optimises the setting, an important factor in the context of psychedelic-related experiences and their therapeutic potential [23, 24]. For instance, when considering neuroplasticity as a therapeutic mechanism of psychedelics, there is evidence that plasticity enhancements can be experience-dependent, suggesting that environmental stimulation may be required to produce measurable plasticity effects [25, 26]. Furthermore, the dose will be determined based on participants’ subjective perception, maximising its therapeutic potential by potentially overcoming individual variation in pharmacokinetics/pharmacodynamics whilst reducing risks of adverse effects that may be due to higher than needed doses/blood concentrations. We will measure self-reflection about microdosing experiences using the phone app journaling system in lieu of integration as an important aspect of psychedelic-based interventions [27]. Overall, our protocol encompasses a similar structure to microdosing psychedelic therapy [23] by allowing the participant to have a preferable intention setting, optimal dose, and reflective integration.

Important concerns are related to compliance and the risk of self-administrating microdoses of LSD at home. To ensure adherence and prevent potential medication stacking, we will use the MDOT procedure to verify dosing events through recorded videos [13, 14]. MDOT was used previously in our laboratory in a trial with a similar design but with a healthy population (MDLSD), confirming 100% compliance with the regimen. No serious adverse events were reported, and other adverse events (e.g. anxiety) were resolved within 2 weeks without the need of medical or psychiatric supervision [13]. In line with these results, our experience with the LSDDEP1 pilot trial [14], although not yet published, shows excellent trial compliance and tolerability.

In addition to compliance and tolerability results, the experience with LSDDEP1, as anticipated, led us to perform adjustments for LSDDEP2 towards increasing screening efficiency, recruitment diversity, speeding up titration, and improving overall data collection. Specifically, for LSDDEP2, we added a re-screening protocol for those who were not onboarded in the trial within the planned time frame. We will accept people of child-bearing potential without a highly effective method of contraception, provided they are not heterosexually active. Titration will be incremented/decremented by 1 or 2 μg as opposed to only 1 μg, and the titration limit will be increased to 20 μg. The onsite dosing will be on the ninth dose as opposed to the first dose, allowing relevant biomarkers to be collected when the participant has reached their “therapeutic” titrated dose. Other minor modifications included adjustments towards reducing the time commitment to take part in the trial, such as having the dispensing of the IMP always done during the data collection session.

We present a rigorously, triple-blinded randomised controlled-placebo trial that will allow us to investigate the possibly superiority of LSD over placebo in a sample of patients with MDD. Regardless of the outcomes, the scientific community will benefit from the results of this trial. If microdosing does not present superiority over placebo, as results from several uncontrolled studies suggest [28, 29], patients would then likely benefit from returning to evidence-based interventions. However, if antidepressant effects are confirmed, then regulated psychedelic compounds could be further developed as medicines, adding a new treatment option for MDD.

## Trial status

The LSDDEP2 trial protocol is currently on version 3.0 (13 December 2023). Recruitment for this trial has commenced on March 2024 under the protocol version 3.0 and is planned to finish by the end of 2025.

### Supplementary Information

Additional file 1: SPIRIT Checklist for Trials.

Additional file 2: Participant Information Sheet.

Additional file 3.

## Data Availability

The corresponding author will release documentation including PIS, consent forms, and study advertisements on publication of trial results. Access to the final trial dataset will only be available to the study investigators and any other relevant regulatory bodies.

## Abbreviations

AE: Adverse event
AUDIT: Alcohol Use Disorders Identification Test
BDNF: Brain-derived neurotrophic factor
BFI-2: Big Five Inventory-2
BMI: Body mass index
CEQ: Credibility and Expectancy Questionnaire
CFI: Cognitive Flexibility Inventory
CONSORT: Consolidated Standards of Reporting Trials
CRF: Case report form
C-SSRS: Columbia-Suicide Severity Rating Scale
DARS: Dimensional Anhedonia Rating Scale
DASS21: Depression Anxiety Stress Scales
DESS: Discontinuation-Emergent Signs and Symptoms
DMT: N,N-Dimethyltryptamine
DSM-5: Diagnostic and Statistical Manual 5
ECG: Electrocardiogram
eCRF: Electronic case report form
EEG: Electroencephalogram
FFMQ: Five Facets of Mindfulness Questionnaire
GMP: Good Manufacturing Practice
HAM-A: Hamilton Anxiety Rating Scale
HAMD-6: Hamilton Rating Scale for Depression
ICH GCP: International Conference on Harmonisation Good Clinical Practice
IMP: Investigational Medicinal Product
LDAEP: Loudness Dependence of Auditory Evoked Potentials
LSD: Lysergic acid diethylamide
LTP: Long-term potentiation
MADRS: Montgomery-Åsberg Depression Rating Scale
MDLSD: Randomised, double-masked, placebo-controlled trial of repeated microdoses of LSD in healthy volunteers
MDOT: Mobile Directly Observed Therapy
MedDRA: Medical Dictionary for Regulatory Activities
MMN: Mismatch negativity
MODTAS: Modified Tellegen Absorption Scale
mRNA: Messenger ribonucleic acid
NM-ASSIST: NIDA (National Institute of Drug Abuse) Modified Alcohol, Smoking and Substance Involvement Screening Test
PIS: Participant information sheet
PK: Pharmacokinetic
PTSD: Post-traumatic stress disorder
REDCap: Research Electronic Data Capture
SAE: Serious adverse event
SAP: Statistical analysis plan
SNPs: Single-nucleotide polymorphisms
TSC: Trial steering committee
VAS: Visual analogue scales
WHOQOL-BREF: World Health Organization Quality of Life Brief Version
WSC: Watts Connectedness Scale
