# Supplementary material for: LSDDEP2: study protocol for a randomised, double-dummy, triple-blind, active placebo-controlled, parallel groups trial of LSD microdosing in patients with major depressive disorder
Source: Trials. 2024 Aug 24;25:560. doi: 10.1186/s13063-024-08384-3 (PMC11344334; doi:10.1186/s13063-024-08384-3)
Supplement: Supplementary file 2 — Additional file 2: Participant Information Sheet. [file 13063_2024_8384_MOESM2_ESM.pdf]

The University of Auckland
Private Bag 92019
Auckland
New Zealand

Building 505, Level 3, 85 Park Road, Grafton
Auckland 1142, New Zealand

Telephone: 64 09 9232787
Email: sd.muthu@auckland.ac.nz

Participant Information Sheet

Assessing the effects of LSD microdosing in people experiencing depression (LSDDEP2)

Formal Study title: A randomised, double-dummy, triple-blind, active placebo-controlled, parallel groups, trial of LSD microdosing in patients with major depressive disorder (LSDDEP2).

Sponsor name and address: The University of Auckland, Auckland 1023, New Zealand.

Lead Researcher: Associate Professor Suresh Muthukumaraswamy

Study Site: Clinical Research Centre, Building 507, Grafton Campus.

Contact phone number: 64 09 9232787

Ethics committee ref.: 2022 FULL 13536

You are invited to take part in a study investigating the use of microdoses of LSD as a treatment for major depressive disorder. Whether or not you take part is your choice. If you don't want to take part, you don't have to give a reason, and it won't affect the care you receive. If you do want to take part now, but change your mind later, you can pull out of the study at any time.

This Participant Information Sheet will help you decide if you'd like to take part. It sets out why we are doing the study, what your participation would involve, what the benefits and risks to you might be, and what would happen after the study ends. We will go through this information with you and answer any questions you may have. You do not have to decide today whether you will participate in this study. Before you decide you may want to talk about the study with other people, such as family, whānau, friends, or healthcare providers. Feel free to do this.

This document is 20 pages long, including the Consent Form. **Please make sure you have read and understood all the pages.**

If you agree to take part in this study, you will be asked to sign the Consent Form on the last page of this document. You will be given a copy of both the Participant Information Sheet and the Consent Form to keep.

VOLUNTARY PARTICIPATION AND WITHDRAWAL FROM THIS STUDY

It is up to you if you take part in this study or not. If you do decide to take part, you will be given this information sheet to keep and be asked to sign a consent form. If you don't want to take part, you don't have to give a reason. If you decide to take part, you are still free to withdraw at any time and without giving a reason. A decision to withdraw at any time, or a decision not to take part, will not affect the standard of care you receive from us or your participation in future studies.

If you take part in the study you have the right to access any information about you collected during the study after your completion or withdrawal from the study.

If we learn anything about your health status or the medications to be tested during the study during the study that affects your health you will be informed of this.

WHAT IS THE PURPOSE OF THE STUDY?

Depression is one of the most common health issues in New Zealand. Unfortunately, current treatments for depression be they drugs or talk therapy don't work for many patients and/or have unpleasant side-effects. There are many anecdotal claims that taking very small doses of LSD can help relieve symptoms of depression but there is no clinical trial evidence to support these claims. Taking small doses of LSD is called microdosing. Unlike large doses of LSD, microdoses will not make you "trip". The goal of our research is to test whether taking microdoses of LSD can help people who are experiencing depression. This would be the first step in testing whether LSD microdosing could be a new potential treatment for depression. LSD is a Class A controlled substance ant approved for use in New Zealand for any medical condition.

HOW IS THE STUDY DESIGNED?

This study aims to recruit 90 individuals with major depressive disorder. This study is a randomised, placebo-controlled double-blinded trial. Randomised means that half of the participants will receive LSD microdoses and half will receive a placebo. The placebo for this trial will be either caffeine or ritalin. Double-blinded means that neither the study team nor the participants will know who receives what. This is to prevent bias in the trial. Unless there is an emergency we will not "de-blind" the trial until the trial is completed. As such, it may take several years before you find out what you received. We will notify you when it is finished and tell you your allocation.

To participate you will have to undergo a psychological and physical evaluation to ensure you meet the inclusion and exclusion criteria (listed below under "Who can take part in this study?"). This will be done in two parts.

- The first part will be done by video call or in person to perform the psychological evaluation. This will take approximately 90 minutes. If you are still eligible, then you will be invited to attend our study site for a screening visit. At that assessment we will take a number of physical measurements, including blood samples, urine samples to check for recreational drug use, blood pressure measurements and an electrocardiogram (ECG) (recordings from your heart). This will take approximately 90 minutes.
- If you are still eligible you will receive a phone call inviting you to attend your first study visit. This visit will take approximately three hours. In this session we will record your

EEG (brain waves - see below for more information and a picture of what an EEG is) and ask you to complete a number of psychological questionnaires and cognitive tests and give another blood sample.

- After this session you will then microdose at home for eight weeks taking a further 15 doses in this period. You will take two microdoses a week. We will provide you up to eight microdoses at a time. During these eight weeks you will complete a questionnaire every night which includes a health check. Every two weeks during this period we will call you and conduct a brief psychological assessment.
- For your ninth dose (start of fifth week) we will ask you to visit our laboratory to take the dose there instead of taking it at home. This visit will take approximately seven hours. During this "Dosing" session we will take a number of blood samples and an EEG will be performed during as well as more questionnaires to complete.
- At the end of the eight weeks there will be a follow-up ("Measure") visit that will take four hours. We will take more blood samples, record another EEG, ECG and ask more questionnaires and do an interview. This is the end of the first part of the study and takes about 20 hours.

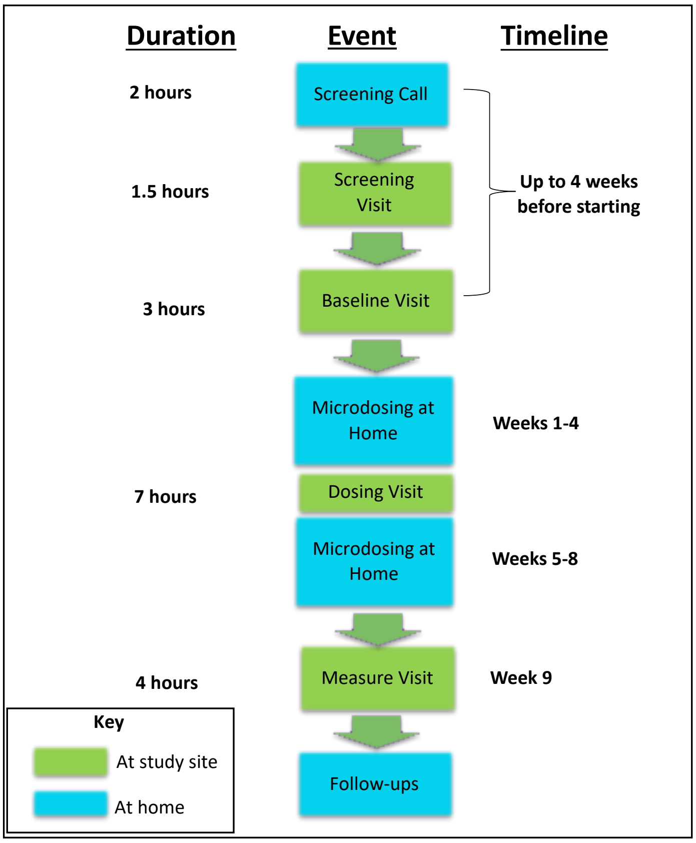

- After the "Measure" period all participants will be offered entry into an <u>open-label extension</u> period. Should you wish, you can then enter the extension period where you will receive eight weeks of LSD microdoses. There is no placebo in the open-label extension and you <u>will</u> receive LSD microdoses during this extension period. The extension period will involve two further visits – repeating the "Dosing" and "Measure" sessions and the microdosing pattern in between. The extension period will take another 11 hours of your time.
- We will then conduct follow-up phone calls, 1, 3 and 6 months after your last visit. A more detailed description of the study timeline and procedures is provided in the "What will my participation involve section".
- **You are welcome to bring a family/whānau member or other support person to any clinic visit.**

WHO CAN TAKE PART IN THE STUDY?

You have asked to participate in this study because you have indicated that you have depression. You must be aged between 21 and 65 years, and not be treatment resistant to two or more classes of antidepressants to take part. SSRIs are the most common "class" of

antidepressant. Being treatment resistant to a class of antidepressants means that you have tried a particular anti-depressant for a reasonable time for it to work, but it has not worked. Our study medical team will help determine if you meet the criteria for inclusion in this study.

You will not be eligible to participate in this study if you:

- Take certain prescription medicines including some specific antidepressants.
- Have or have had certain medical or mental health conditions other than depression. This includes: schizophrenia, psychosis bipolar disorder, PTSD, eating disorders, kidney, liver or heart/cardiovascular conditions.
- Are feeling suicidal. See "What are the possible risks of this study?" below for more information.
- Have recently had a substance use disorder.
- Are pregnant or breastfeeding, or of child-bearing potential and not on an effective method of contraception. See section "What are the possible risks of this study?" for more information.

During the study we ask that you please:

- Abstain from alcohol for the 24 hours before the first LSD microdosing session.
- Abstain from recreational drugs for the duration of the study
- Limit caffeine consumption to 100 mg on a dosing day (about one cup of coffee or two cups of tea).
- Don't engage in any other therapies for depression and remain on any current antidepressant medications for the duration of the study (if applicable).

Please note: the study drug contains small amounts of alcohol (~ 1ml) that is given under your tongue. For some people will taste unpleasant or they may not wish to consume any alcohol. Let us know if this will be a problem for you.

WHAT WILL MY PARTICIPATION IN THE STUDY INVOLVE?

Your participation starts with some screening sessions to determine your eligibility to take part in the study. A study team member will contact you by phone/email to set-up the screening process

**SCREENING - up to 28 days before dosing starts**	
Screening<br>(Video call*)<br><br>2 hours	We will ask you questions about your:<br>• Demographics<br>• Medical history<br>• Psychiatric history. This includes you telling us your personal history and us asking you standardised questions<br>You will complete:<br>• Questionnaires about your personality<br>• Questionnaires about your mental health<br>This video call will not be recorded.
If you are still eligible then you will be asked to attend a screening visit in person	
Screening<br>(Visit 1)	We will collect:<br>• Height and weight information

1.5 hours	• Blood samples (25 ml of blood) to test your kidney and liver function and a pregnancy test if required. 25 ml is about 5 teaspoons<br>• Blood pressure and heart rate<br>• Urine drug screen for recreational drugs and a breathalyser test<br>• Electrocardiogram (ECG) recordings of your heart

*This can be done in person if you prefer or don't have access to video call technology

Confirmation of enrolment: Once we have all your test results back, we will contact you to confirm your enrolment and book your next session time. If we are still unsure about eligibility we may contact you to get further information from you.

**BASELINE - 4 to 8 days before dosing starts**	
Baseline Measurements (Visit 2)<br><br>3 hours	This will involve:<br>• An EEG procedure which lasts 90 minutes (see below)<br>• Interviews about your current psychiatric state<br>• Questionnaires about your current mental health<br>• There will be a questionnaire about your sexual function done on an ipad. For your privacy you can complete this questionnaire in a private room.<br>• Measurements of heart rate and blood pressure<br>• Blood samples (15 ml of blood)<br>• Set-up the study app on your mobile phone* (details below)<br>• Set-up the Garmin activity tracker (details below)<br>• A short interview and questionnaire about your expectations<br>Audio from this interview will be recorded. These will be held indefinitely on password protected databases via password protected servers.<br><br>At the end of this day we will provide you your first eight microdoses. You will microdose twice a week. You can choose the days you microdose to suit your schedule as long as there is one day at least between doses.<br><br>From the baseline visit we will ask you to fill out a nightly questionnaire which takes a few minutes each night.<br><br>If you have any health concerns you can use the app to indicate that you would like to talk to a study team member and we will contact you.

*If you don't have an appropriate smart phone or adequate data plan we will provide these for you.

**DOSING PERIOD (Day 1 to 57 - 8 weeks long)**	
8 weeks long including a Dosing Visit<br><br>(Visit 3 7 hours)	During this period you will microdose twice per week. The doses are contained in glass vials so you will draw it up and put an oral syringe (with no needle) under your tongue yourself. The placebo is in capsule form. At each dosing you will also need to take placebo capsules. You may need to take up to 6 placebo capsules at a time. We will provide

	you instructions at the Baseline session and the Study App will help you to self-administer the drugs.<br><br>In the microdosing regimen we have made, we aim to get you to engage in therapeutically helpful activities while you are microdosing. During the baseline session we will discuss potential activities with you and program these as suggestions into the study app. The app will remind you of these activities as you plan your microdosing days.<br><br>Your 9th dose will be taken during a visit to our laboratory. This visit will involve<br>• Being administered a microdose in a monitored environment.<br>• We will take regular measurements of your heart rate, blood pressure, mood state and intermittent blood samples (60 ml of blood)<br>• An EEG procedure which lasts 90 minutes (see below)<br>• Electrocardiogram (ECG) recordings of your heart<br>• Questionnaires about your current mental health<br>• Interview about your current psychiatric state<br>• At this visit we will provide you your second set of microdoses.<br><br>At this visit we will supply you the last seven microdoses.<br><br>At two and six weeks into microdosing we will schedule a video call with you to conduct a short psychiatric interview and have you do some mental health questionnaires.

MEASURE (Day 57 - ~ 8 weeks)	
Measurement Day<br>(Visit 4)<br><br>4 hours	This will involve<br>• An EEG procedure which lasts 90 minutes (see below)<br>• Electrocardiogram (ECG) recordings of your heart<br>• Interviews about your current psychiatric state<br>• Questionnaires about your current mental health<br>• There will be a questionnaire about your sexual function done on an ipad. For your privacy you can complete this questionnaire in a private room.<br>• Measurements of heart rate and blood pressure<br>• Blood samples (40 ml of blood)<br>.

Up to here the study will have involved 4 visits over a 2-3 month period and taken about 20 hours of your time. At this point in the study you can enter the open-label extension period if you wish. Entering the extension period means you can repeat the “Dosing” and “Measurement” sections again. You will receive LSD in the extension period. If you don’t wish to use the extension period we can begin Follow-ups.

Follow-ups (1 month, 3 months, 6 months later)	
Phone calls only<br><br>About 30 minutes each	Follow-ups involve:<br>• Questionnaires about your current mental health<br>• Interviews<br><br>At the end of the one-month follow-up you can stop filling in nightly questionnaires and we will provide you an envelope to return the fitness tracker.

MICRODOSING AT HOME:
Your home microdosing will be guided by the study App. The App will guide you how to measure out each dose and provide instructions on eating/drinking at each administration. We will show you how to use the App at the first Dosing session. The App will get you to video record each dose that you take and will send the videos to us so that we can confirm it is being done correctly. We will delete these videos as soon as we receive and check them.

ECG:
ECG involves putting a number of electrodes around your chest. This is quite close to your breast area. If this makes you uncomfortable let us know. We will do our best to find a study team member of your own gender to perform this procedure or be in the room if preferred - but this is subject to availability of staff.

EEG:
An EEG recording involves putting on a soft cap that has 64 electrodes (black plugs in the above image). The electrodes sit near your scalp and record electrical activity from your brain while you complete simple tasks on a computer. A good electrical signal is reached by using an electrolyte gel to ensure good contact with your skin. After the recording session, the electrolyte gel needs to be removed from the hair, which is easily done by a hair wash. We have facilities for you to wash your hair and will provide you with a towel and shampoo.

The EEG takes around 15-30 minutes to setup, and the simple tasks will take up to an hour and a half (total 90 minutes). To make the set-up easier please come to the study days with clean, dry hair with no hair-care products in your hair.

The EEG procedure consists of a set of easy visual and auditory tasks. One of the tasks is a “gambling task” with a small monetary reward ($20 on average) involved. The EEG recordings we make are not able to detect clinical abnormalities and are made for scientific purposes only.

MEDIA AND SOCIAL MEDIA:
Please don’t post on on social media or talk to media organisations about your involvement in the study until the study is complete (we will let you know when it is finished). This is to prevent your viewpoints influencing those of future trial participants.

WHAT IS YOUR RESEARCH CENTER LIKE?

Here we provide some pictures of some of the main locations and procedures to help you make your choice about participating.

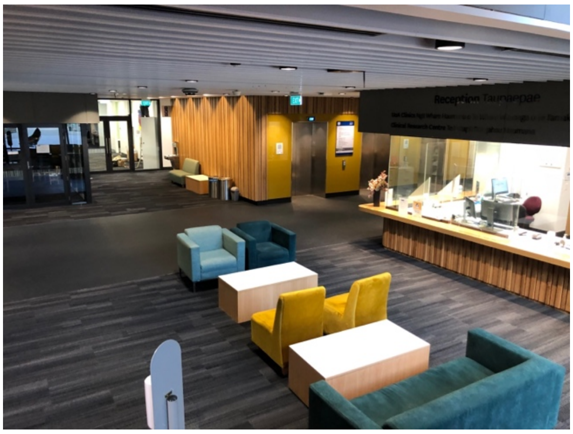

Our research centre reception

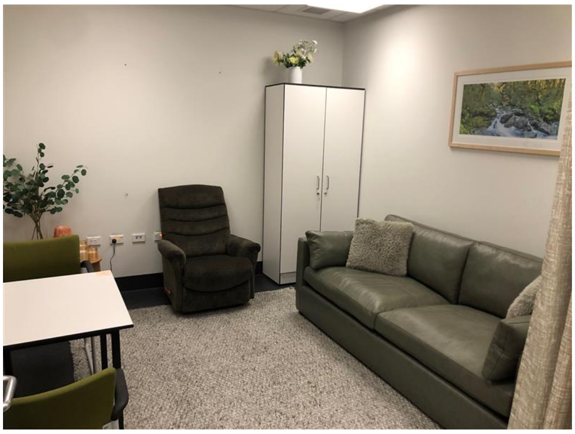

The main research room

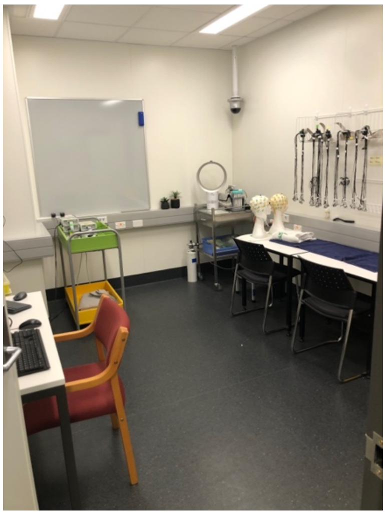

The EEG room

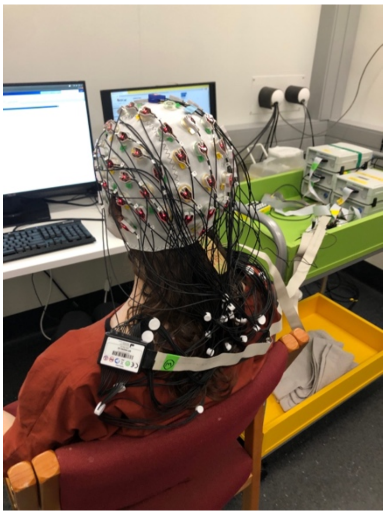

An EEG in progress

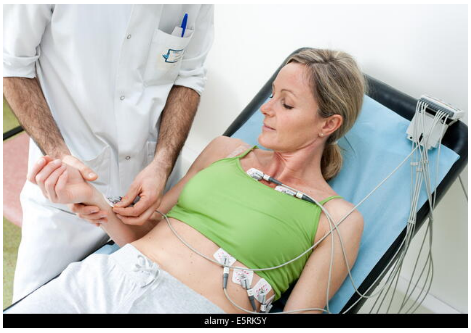

ECG

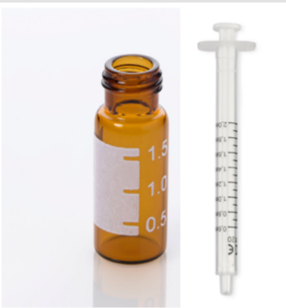

Oral syringe and vial for microdosing

WHAT WILL HAPPEN TO MY BLOOD AND URINE SAMPLES?

The urine samples that you provide us during some visits will be tested for the use of recreational substances and then disposed of immediately.

Blood samples will be collected at various points in the study by the study team. All samples will be de-identified (no name on them) and no samples will be sent overseas.

Some blood samples will be sent to LabPlus for immediate analysis to look at simple health markers (e.g. liver and kidney function) to ensure participants are safe to be involved in the research. If you are sexually active and of child-bearing potential (able to become pregnant), it is very important that you are not pregnant or become pregnant during this study. As such, LabPlus will also conduct a blood pregnancy test. For further information about the study requirements surrounding pregnancy; see below (Possible risks of this study). Only LabPlus will have access to these samples. The samples will be disposed of in accordance with LabPlus policy after they have been analysed. We will inform you of any unexpected results from these tests that could be of importance for you to know and suggest an appropriate course of action.

All other blood samples will be kept stored at The University of Auckland for scientific analysis for up to five years. Should you withdraw from the study your samples will continue to be analysed for the purposes of the study. We will be looking at the blood samples to look at levels of LSD in your body as well as protein, hormonal and inflammatory markers that might get changed by the drug. Any analyses of the samples you provide us will be only for the purposes of microdosing and/or depression research.

You may hold beliefs about a sacred and shared value of all or any tissue samples removed. The cultural issues associated with storing your tissue should be discussed with your family/ whānau as appropriate. There are a range of views held by Māori around these issues; some iwi disagree with storage of samples citing whakapapa and advise their people to consult before participating in research where this occurs. However, it is acknowledged that individuals have the right to choose. Options for disposal of the blood samples with karakia and returning samples can be discussed during the screening visit. If there are additional things that we can do to meet your needs, please feel free to discuss these with the study team

WHAT ARE THE POSSIBLE RISKS OF THIS STUDY?

In the event that a condition which is assessed to be a clinical abnormality is detected through a blood test or other exam you will be informed. Your general practitioner or other health professional of your choice will be notified.

The drug that we will use in this study are given at very low doses. We will be very careful and so your first dose will be given under clinical supervision at our research centre. The LSD has been prepared by a pharmaceutical company under strict manufacturing conditions to ensure the drug is pure.

Some blood samples will be obtained through an intravenous (IV) cannula. An IV cannula is a small flexible tube put into a vein in your arm, the same as if you have been on a drip in a hospital. This can cause side-effects. The most common of these are bruising, mild pain and feeling faint when it is put in.

Previous LSD microdosing studies in healthy volunteers have reported mild/moderate intensity headaches, nausea, vivid dreams, anxiety and jitteriness. You might also experience these. If you do, it is important to let us know so we can advise you. Some participants also reported feeling some stimulation, a bit like you feel after drinking coffee. If you experience this, we have a plan in place to change the dose amount you receive. The amount you receive

at every dose will change every day – this is called a 'titration protocol'. Trial staff will guide you through how this works and the study app will also assist with this procedure. There may be other side-effects from microdosing that we are scientists do not know about yet as there have not been any studies in depressed patients yet.

The placebos for this trial are caffeine and ritalin. These have been chosen to mimic the stimulant effects of LSD microdoses. The side-effects of caffeine are well known and include feeling over-stimulated/jittery. The side-effects of ritalin can include nervousness, overstimulation, hallucinations, blurred vision and nausea (see https://www.medsafe.govt.nz/profs/datasheet/r/RitalintabSRtabLAtab.pdf).

Although the doses of all drugs given are very low and should not have any psychedelic effects, we advise that you should not drive, operate machinery, or engage in any dangerous activities or important care giving roles where you could put yourself or others in harm's way for six hours after taking a dose. Do not change these plans, even if you do not feel any effects or have not felt any effects so far as your reaction to each dose might not be the same each time, so it is essential that you maintain these arrangements for every dose day.

You should be aware that LSD is a Class A controlled substance. Although given at very low doses, taking this regularly may be incompatible with your employment conditions. You should discuss this with your employer and/or the study team before starting the study. LSD is a Class A controlled substance and remains the property of the University of Auckland and must be returned if requested and should only be used as directed. Failure to do these things may be a criminal offence and the New Zealand police may be contacted.

It is important that you keep the study drugs safe, out of the reach of children and you should not share them with others. If required, we can provide you with a lockbox to keep your home doses safe. You should take the doses exactly as requested. It may be unsafe for you to not do so. Please discuss this with the study team.

For participants currently taking antidepressant medication, the interaction between psychedelics and antidepressants is currently unknown. Recent evidence looking at this interaction effect suggests there may be minimal effects, however this type of research is still in its early stages. It is theoretically possible that LSD and antidepressants used together might cause "serotonin syndrome" which can cause shivering, fever, muscle rigidity and even seizures. However, as this study involves taking very small doses of LSD we do not expect to see any negative interactions. Your safety for participation will be established by our study physician at screening.

As a part of this research, we will ask you to complete a number of psychological questionnaires. These include questionnaires that ask about depression and suicide. If these cause you distress our research team is available to support you or refer you to someone else who can.

We have a number of safety protocols in place to check and monitor your well-being during the study. We monitor the nightly questionnaires you complete every day. If you raise any health issues we will follow these up the next day. Similarly, when we call you every two weeks we will check in on your general well-being. If you have any questions about this, please feel

free to speak with your doctor or with study staff. We are happy to answer any questions you might have.

Reproductive risks for heterosexually active participants of child-bearing potential

The effects of LSD in pregnancy and breastfeeding are unknown, but there is a risk it may cause birth defects or foetal deaths, and/or be passed on in breast milk. If you are pregnant or breastfeeding, you cannot take part in this study.

If you are sexually active and of child-bearing potential (able to become pregnant), it is very important that you do not become pregnant during this study. You must use one of the methods of contraception listed below, from at least one moth before your first dose of study drug until at least one month after your last dose:

A highly effective method (less than 1 pregnancy per 100 women using the method for one year) e.g.:

- Implant contraceptive (e.g., Jadelle®)
- Intra-uterine device (IUD) containing either copper or levonorgestrel (e.g., Mirena®)
- Male sterilization (vasectomy)
- Female sterilisation (e.g., bilateral tubal ligation ('clipping or tying tubes') or hysterectomy)

OR an effective method (5 - 10 pregnancies per 100 women using the method for one year) e.g.:

- Injectable contraceptive (e.g., Depo Provera)
- Oral Contraceptive Pill (combined hormonal contraceptive pill or progestogen-only 'mini-pill')

You must also agree not to donate eggs, from dosing until at least 3 months after your last dose of study drug.

If you do become pregnant during the study, you must tell a member of the study team as soon as possible. If you are pregnant, this will result in the cessation of the study for you and we will ask to collect information about the pregnancy and outcomes, including that of the infant.

Reproductive risks for heterosexually active participants able to father a child

The effects of LSD if passed on through semen are unknown, but there is a risk it may cause birth defects or foetal deaths. You are responsible for informing your sexual partner of these possible risks.

If you are heterosexually active and have any partner who is of child-bearing potential (meaning a partner who may become pregnant) it is very important that you use contraception during this study. You and your partner must use one of the contraception options listed above for participants of child-bearing potential, from at least 10 days before your first dose of study drug through until at least 72 hours after your last dose.

If a pregnancy occurs, you must report this to a member of the study team as soon as possible. Your partner will be asked to give consent for their information and their infant's information to be collected for monitoring purposes.

You must also agree not to donate sperm, from dosing until at least 3 months after your last dose of the study drug.

WHAT ARE THE POSSIBLE BENEFITS OF THIS STUDY?

You may experience a reduction in depressive symptoms. You will be informed of any new information about beneficial effects related to LSD microdosing that becomes available during the study that may have an impact on your health.

The results from this study may help advance knowledge on the biology of depression and open new avenues of treatment to help people with depression in the future.

WHAT ARE THE ALTERNATIVES TO TAKING PART?

You do not have to participate in this study to receive treatment for your depression. There are other treatments for depression such as medications and talk therapies that are known to be effective. These can be discussed with your doctor.

WILL ANY COSTS BE REIMBURSED?

We will pay for any costs that you incur by taking part in the study. If you require a taxi to get to and from the study, then we can arrange and pay for this. We recognise that taking part in the study will take up a lot of your time. The first part of the study will take up to 20 hours of your time. We will provide you with a minimum of $250 as koha. An extra $20 can be gained based on results from one of the EEG tasks which is performed three times. If you choose to continue into the extension period, which takes a further eleven hours of time we will not provide further koha for this time, except for extra money based on results from one of the EEG tasks as described above.

WHAT IF SOMETHING GOES WRONG?

As this research study is for the principal benefit of its commercial sponsor The University of Auckland if you are injured as a result of taking part in this study you **won't** be eligible for compensation from ACC.

However, The University of Auckland has satisfied the Southern Health and Disability Ethics Committee that approved this study that it has up-to-date insurance for providing participants with compensation if they are injured as a result of taking part in this study.

New Zealand ethical standards require compensation for injury to be at least ACC equivalent. Compensation should be appropriate to the nature, severity and persistence of your injury and should be no less than would be awarded for similar injuries by New Zealand's ACC scheme.

Some sponsors voluntarily commit to providing compensation in accordance with guidelines that they have agreed between themselves, called the Medicines New Zealand Guidelines (Industry Guidelines). These are often referred to for information on compensation for commercial clinical trials. There are some important points to know about the Industry Guidelines:

- On their own they are not legally enforceable and may not provide ACC equivalent compensation.
- There are limitations on when compensation is available, for example compensation may be available for more serious, enduring injuries, and not for temporary pain or discomfort or less serious or curable complaints.

- Unlike ACC, the guidelines do not provide compensation on a no-fault basis:
- The Sponsor may not accept the compensation claim if:
  - Your injury was caused by the investigators, or;
  - There was a deviation from the proposed research plan, or;
  - Your injury was caused solely by you.

An initial decision whether to compensate you would be made the by the sponsor and/or its insurers.

If they decide not to compensate you, you may be able to take action through the Courts for compensation, but it could be expensive and lengthy, and you might require legal representation. You would need to be able to show that your injury was caused by participation in the trial.

You are strongly advised to read the Industry Guidelines and ask questions if you are unsure about what they mean for you.

If you have private health or life insurance, you may wish to check with your insurer that taking part in this study won't affect your cover.

WHAT WILL HAPPEN TO MY INFORMATION?

During this study, the study doctors/researchers, nurses and other study staff will record information about you and your study participation. This includes the results of any study assessments. If needed, information from your hospital records and your GP may also be collected; access to your records is limited to that required for study purposes.. You cannot take part in this study if you do not consent to the collection of this information.

<u>Identifiable Information</u>

Identifiable information is any data that could identify you (e.g. your name, date of birth, or address, video/audio recordings). The following groups may have access to your identifiable information:

- University of Auckland investigators, staff and PhD students (to complete study assessments).
- Study monitors, to make sure the study is being run properly and that the data collected is accurate.
- Your GP may be notified of your participation in this study with your consent.
- University of Auckland representatives, <u>if</u> you make a compensation claim for study-related injury. Identifiable information is required in order to assess your claim.
- University of Auckland representatives, ethics committees, or government agencies from New Zealand or overseas, <u>if</u> the study or site is audited. Audits are done to make sure that participants are protected, the study is run properly, and the data collected is correct.
- Your usual doctor (your GP or specialist), if a study test gives an unexpected result that could be important for your health or well-being. This allows appropriate follow-up to be arranged.
- Rarely, it may be necessary for a study doctor to share your information with other people – for example, if there is a serious threat to public health or safety, or to the life or health of you or another person or if the information is required in certain legal situations.

- As a part of this research, we will need you to video record you taking the microdoses to ensure that the dosing instructions are being followed. These video are deleted after they are reviewed by the study team. No other video recordings are taken during the study.
- As part of the research we will record audio of some your psychiatric assessments, for quality checking purposes), audio journals if you choose to keep audio journals and speech recordings on Dosing day. These audio recordings will be used for scientific analysis. Access to these audio recordings is limited to the groups mentioned above and may be stored for up to fifteen years.

De-identified (Coded) Information

To make sure your personal information is kept confidential, information that identifies you will not be included in any report generated by the study team. The study team will keep a list linking your code with your name, so that you can be identified by your coded data if needed.

The following groups may have access to your coded information which may be sent and stored overseas:

- People and companies working with or for the University of Auckland, for the purposes of this study (this may include approximately 20 people and companies).
- Regulatory or other governmental agencies worldwide.

The results of the study may be published or presented, but not in a form that would reasonably be expected to identify you.

Anonymised Information.

The University of Auckland may remove the code from your de-identified information – this is called 'anonymisation'. This makes it very difficult (but not impossible) to identify the information that belongs to you. The University of Auckland may share this anonymised information with other researchers and companies.

Future Research Using Your Information.

Your de-identified information may be used for future research related to microdosing or depression.

This future research may be conducted overseas. You will not be told when future research is undertaken using your information. Your information may be shared widely with other researchers or companies. Your information may also be added to information from other studies, to form much larger sets of data.

You will not get reports or other information about any / some research that is done using your information.

Your information may be used indefinitely for future research unless you withdraw your consent. However, it may be extremely difficult or impossible to access your information, or withdraw consent for its use, once your information has been shared for future research.

Security and Storage of Your Information.

Your identifiable information is held at The University of Auckland during the study. After the study it is stored for at least fifteen years. Your coded information will be entered into electronic case report forms. Coded study information will be kept by the University of Auckland in

secure, cloud-based storage indefinitely. All storage will comply with local and/or international data security guidelines.

Risks.

Although efforts will be made to protect your privacy, absolute confidentiality of your information cannot be guaranteed. Even with coded and anonymised information, there is no guarantee that you cannot be identified. The risk of people accessing and misusing your information (e.g. making it harder for you to get or keep a job or health insurance) is currently very small, but may increase in the future as people find new ways of tracing information.

Your coded information may be sent overseas. Other countries may have lower levels of data protection than New Zealand. There may be no New Zealand representation on overseas organisations which make decisions about the use of your information. There is a risk that overseas researchers may work with information in a way that is not culturally appropriate for New Zealanders.

This research includes basic information such as your ethnic group, geographic region, age range, and gender. It is possible that this research could one day help people in the same groups as you. However, it is also possible that research findings could be used inappropriately to support negative stereotypes, stigmatise, or discriminate against members of the same groups as you.

Rights to Access Your Information.

You have the right to request access to your information held by the research team. You also have the right to request that any information you disagree with is corrected.

Please ask if you would like to access the results of your screening and safety tests during the study. You may access other study-specific information before the study is over, but this could result in you being withdrawn from the study to protect the study's scientific integrity.

If you have any questions about the collection and use of information about you, you should ask the study team.

Rights to Withdraw Your Information.

You may withdraw your consent for the collection and use of your information at any time, by informing a study team member.

If you withdraw your consent, your study participation will end, and the study team will stop collecting information from you.

Information collected up until your withdrawal from the study will continue to be used and included in the study. This is to protect the quality of the study.

Ownership Rights.

Information from this study may lead to discoveries and inventions or the development of a commercial product. The rights to these will belong to The University of Auckland and MindBio Therapeutics Ltd. You and your family will not receive any financial benefits or compensation, nor have any rights in any developments, inventions, or other discoveries that might come from this information.

Use of New Technologies (Mobile phone App and Activity Tracking)

In this study use of a mobile phone application and Garmin activity tracker are mandatory components for study participation. We will provide you with the Activity tracker and if you do

not have a mobile phone or data plan we will provide you with one or both as needed at no cost to you.

The mobile phone app has been written by researchers at The University of Auckland. It is designed to collect questionnaire data from you, has a calendar function to help remind you of visits, help you log adverse effects and provide other information about the study for you so you can access it easily. The study app collects videos of your dosing and audio diaries if you choose to use them. These are identifiable information. The app will send data directly only to our secure study database where the rest of your data is held.

We will ask you to wear a Garmin Activity tracker for the duration of the study as we are interested in recording your activity and sleep during the study. These data will be sent to the Garmin website and to an associated company called Fitrockr.

In order to protect your privacy we will make dummy email addresses and accounts to associate with the watch that you will wear so that your personal information does not need to be registered with Garmin/Fitrockr. Before we give you the watch we will turn the GPS function off so that neither the research team nor anyone else can track your location. We would ask you not to turn the GPS functions on. Only the research team and you have access to your dummy account and password.

Although your activity tracker data is “de-identified” you should note that your data will be stored overseas in both the United States for Garmin and in Germany for FitRockr. These companies may use your de-identified data for their own purposes beyond the aims of our study. Although these companies require consent to share your de-identified data with third parties we are unable to guarantee this is the case.

There are no costs to you involved with using the watch and its services. We will need to install the Garmin app on your phone so that your data can be transmitted from the watch to your phone to the Garmin/Fitrockr overseas data clouds. We will access your data from there. We will ask you to return the Activity tracker to us at the end of the study.

The full data policies of these companies are at these links:

https://www.fitrockr.com/health-solutions/privacy-policy/

https://www.garmin.com/en-NZ/privacy/connect/policy/#categoriesOfPersonalDataProcessedByGarmin

Māori Data Sovereignty

Māori data sovereignty *is* about protecting information or knowledge that is about (or comes from) Māori people. We recognise the taonga of the data collected for this study. To help protect this taonga:

- We have consulted with a Māori research advisors about the collection, ownership, and use of study data.
- We will allow Māori organisations to access de-identified study data, for uses that may benefit Māori.
- We have conducted several hui regarding the study, included several Māori patients in the design process and will continue to do so as the study advances.

WHAT HAPPENS AFTER THE STUDY OR IF I CHANGE MY MIND?

The medication that you receive during the study will not be available to you after your participation in this study, as it is not approved for the treatment of depression in New Zealand. LSD is a controlled Class A drug in New Zealand.

You may withdraw your consent for the collection and use of your information at any time, by informing a member of the study team. If you withdraw your consent, your study participation will end, and the study team will stop collecting information from you. Information collected up until your withdrawal from the study will continue to be used and included in the study. This is to protect the quality of the study.

Can I find out the results of the study?

It can take quite a long time to analyse data from these kinds of studies. We hope to be able to tell you the final results one to two years after completion of the study. We plan to publish the results in specialised academic journals. If you want us to, we can send you a summary of the results in an easier format to read.

This trial is registered on the Australian New Zealand Clinical Trials Registry (ANZCTR). This can be accessed at anzctr.org.au

Who is funding the study?

The Health Research Council (HRC) of New Zealand is funding the trial. Funding for the study drug and study app has been provided by a company called MindBio Therapeutics Ltd. The study investigators are all affiliated with The University of Auckland and/or work for private healthcare providers in New Zealand. The University of Auckland has a licensing agreement in place with MindBio Therapeutics Ltd. and both parties may benefit commercially from the study being conducted. The data in this study will be included in several student PhD theses.

Who Has Approved the study?

This study has been approved by an independent group of people called a Health and Disability Ethics Committee (HDEC), who check that studies meet established ethical standards. The Southern Health and Disability Ethics Committee has approved this study.

The scientific aspects of this study have been approved by the Standing Committee on Therapeutic Trials (SCOTT), which is part of Medsafe. Medsafe have also approved the pharmaceutical quality of the study drug.

Who do I contact for more information or if I have concerns?

If you have any questions, concerns or complaints about the study at any stage, you can contact:

Dr Suresh Muthukumaraswamy, Associate Professor
Phone: +64 9 9232787
Email: sd.muthu@auckland.ac.nz

For urgent questions and concerns that arise during the study you can contact:

On-call (24/7) study team member

Phone: 0273107137

If your depression symptoms become severe or you are experiencing suicidal thoughts you can contact:

The Auckland Mental Health Crisis team
Phone: 0800 800 717 (operating 24/7)

If you are in immediate danger, please contact 111 and ask for Police.

If you want to talk to someone who isn't involved with the study, you can contact an independent health and disability advocate on:

If you want to talk to someone who isn't involved with the study, you can contact an independent health and disability advocate on:

Phone:	0800 555 050
Fax:	0800 2 SUPPORT (0800 2787 7678)
Email:	advocacy@advocacy.org.nz
Website:	https://www.advocacy.org.nz/

For Māori health support please contact:
He Kamaka Waiora (Māori Health Team)
Phone: +64 9 486 8324 x 2324
Email: hkw@adhb.govt.nz

You can also contact the health and disability ethics committee (HDEC) that approved this study on:

Phone:	0800 4 ETHIC
Email:	hdecs@health.govt.nz

THE UNIVERSITY OF AUCKLAND
Te Whare Wānanga o Tāmaki Makaurau
NEW ZEALAND

The University of Auckland
Private Bag 92019
Auckland
New Zealand

Building 505, Level 3, 85 Park Road, Grafton
Auckland 1142, New Zealand

Telephone: 64 9232787
Email:
sd.muthu@auckland.ac.nz

Consent Form

Assessing the effects of LSD microdosing in people experiencing depression (LSDDEP2)

Please tick to indicate you consent to the following

I have read the Participant Information Sheet, or have had it read to me in a language I understand, and I fully comprehend what it says.	
I have been given sufficient time to consider whether or not to participate in this study.	
I have had the opportunity to use a legal representative, whanau/ family support or a friend to help me ask questions and understand the study.	
I am satisfied with the answers I have been given regarding the study and I have a copy of this consent form and information sheet.	
I understand that taking part in this study is voluntary (my choice) and that I may withdraw from the study at any time without this affecting my medical care.	
I consent to the research staff collecting and processing my information, including information about my health.	
I consent to my information being sent overseas.	
If I decide to withdraw from the study, I agree that the information collected about me up to the point when I withdraw may continue to be processed.	
I consent to my GP or current provider being informed about my participation in the study and of any significant abnormal results obtained during the study.	
I understand that there may be risks associated with the drug in the event of myself or my partner becoming pregnant. I undertake to inform my partner of the risks and to take responsibility for the prevention of pregnancy.	
I agree to my blood and urine samples being collected and I am aware that these samples will be disposed of using established guidelines for discarding biohazard waste.	
I agree to an approved auditor appointed by the New Zealand Health and Disability Ethics Committees, or any relevant regulatory authority or their approved representative reviewing my relevant	

medical records for the sole purpose of checking the accuracy of the information recorded for the study.	
I understand that my participation in this study is confidential and that no material, which could identify me personally, will be used in any reports on this study.	
I understand the compensation provisions in case of injury during the study.	
I know who to contact if I have any questions about the study in general.	
I understand that LSD is a Class A controlled substance and remains the property of the University of Auckland and must be returned if requested and should only be used as directed. I understand that failure to do these things may be a criminal offence and the New Zealand police may be contacted	
I will take the drugs as directed, will keep them out of reach of children and will not provide them to any other persons and not engage in dangerous activities (like driving) for six hours after taking drug doses.	
I understand my responsibilities as a study participant.	
I wish to receive a summary of the results from the study.	Yes ☐ No ☐

Declaration by participant:
I hereby consent to take part in this study.

Participant's name: ______________________________

Signature: ______________________ Date: ______________

Declaration by member of research team:

I have given a verbal explanation of the research project to the participant and have answered the participant's questions about it.

I believe that the participant understands the study and has given informed consent to participate.

Researcher's name: ______________________________

Signature: ______________________ Date: ______________
