# Supplementary material for: LSDDEP2: study protocol for a randomised, double-dummy, triple-blind, active placebo-controlled, parallel groups trial of LSD microdosing in patients with major depressive disorder
Source: Trials. 2024 Aug 24;25:560. doi: 10.1186/s13063-024-08384-3 (PMC11344334; doi:10.1186/s13063-024-08384-3)
Supplement: Supplementary file 3 — Additional file 3. [file 13063_2024_8384_MOESM3_ESM.pdf]

Health and Disability Ethics Committees

Health and Disability Ethics Committees
Ministry of Health
133 Molesworth Street
PO Box 5013
Wellington
6011
hdecs@health.govt.nz

Ethics reference: 2022 FULL 13536

14 December 2022

Associate Professor Suresh Muthukumaraswamy

85 Park Rd
Grafton
Auckland
1023
New Zealand

Tēnā koe Associate Professor Muthukumaraswamy

APPROVAL OF APPLICATION

Study title: Assessing the effects of LSD microdosing in patients with major depressive disorder (LSDDEP)

I am pleased to advise that your application was **approved** by the Southern Health and Disability Ethics Committee (the Committee) with non-standard conditions. This decision was made through the FULL pathway.

Conditions of HDEC approval

HDEC approval for this study is subject to the following conditions being met prior to the commencement of the study in New Zealand. It is your responsibility, and that of the study's sponsor, to ensure that these conditions are met. No further review by the Southern Health and Disability Ethics Committee is required.

Standard conditions:

- Before the study commences at *any* locality in New Zealand, all relevant regulatory approvals must be obtained.
- Before the study commences at *any* locality in New Zealand, it must be registered in a clinical trials registry. This should be a registry approved by the World Health Organization (such as the Australia New Zealand Clinical Trials Registry, www.anzctr.org.au or https://clinicaltrials.gov/).
- Before the study commences at *each given* locality in New Zealand, it must be authorised by that locality in Ethics RM. Locality authorisation confirms that the locality is suitable for the safe and effective conduct of the study, and that local research governance issues have been addressed.

Non-standard conditions:

Participant Information Sheet addendum for genetic research:

- please expand on the explanation of what DNA is in lay terms.
- please state if extra samples will need to be taken.
- please include a statement on what will happen to my samples.
- please check for spelling errors and general typos.

Non-standard conditions must be completed before commencing your study, however, they do not need to be submitted to or reviewed by HDECs.

If you would like an acknowledgement of completion of your non-standard conditions you may submit a post approval form amendment through the Ethics Review Manager. Please clearly identify in the amendment form that the changes relate to non-standard conditions and ensure that supporting documents (if requested) are tracked/highlighted with changes.

For information on non-standard conditions please see paragraphs 125 and 126 of the *Standard Operating Procedures for Health and Disability Ethics Committees (SOPs)*.

After HDEC review

Please refer to the *SOPs* for HDEC requirements relating to amendments and other post-approval processes.

Your next progress report is due by 14 December 2023.

As your study is an intervention study involving a new medicine, all progress reports **must** be accompanied by an annual safety report. While there is no prescribed format for annual safety reports, they must be no longer than two pages in length, written in lay language, and include a brief description and analysis of:

- new and relevant findings that may have a significant impact on the safety of participants
- the safety profile of the new medicine and its implications for participants, taking into account all safety data as well as the results of any relevant non-

clinical studies
- the implications of safety data to the risk-benefit ratio for the intervention study, and whether study documentation has been or will be updated
- any measures taken or proposed to minimise risks. (Where such a proposed measure would be a substantial amendment, it must be submitted for HDEC review in the normal way).

For the avoidance of doubt, Development Safety Update Reports may serve as annual safety reports to HDECs provided that they contain the information outlined above. These summaries should be accompanied by comment from the New Zealand coordinating investigator of the study.

Please refer to paragraphs 206 to 208 of the *SOPs* for further information.

Participant access to compensation

This clinical trial is to be conducted principally for the benefit of the manufacturer or distributor of the medicine or item being trialled. Section 32 of the Accident Compensation Act 2001 provides that participants injured as a result of treatment received as part of this trial will **not** be eligible for publicly-funded compensation through the Accident Compensation Corporation.

Further information and assistance

Please contact the HDECs Secretariat at hdecs@health.govt.nz or visit our website at www.ethics.health.govt.nz for more information, as well as our General FAQ and Ethics RM user manual.

Nāku noa, nā

Mr Dominic Fitchett

Chair

Southern Health and Disability Ethics Committee

Encl: Appendix A: documents submitted

Appendix B: statement of compliance and list of members

Appendix A: Documents submitted

Document Type	File Name	Date	Version
CV for Coordinating Investigator	HRC NZ standard CV template_Suresh_20210622	06/09/2022	1
Investigator's Brochure	LSD_IB_UOA_V2_13092022	13/09/2022	2.00
Protocol	LSDDEP_Protocol_20220915	15/09/2022	1.0
Data and Tissue Management Plan	LSDDEP_DataManagementPlan_20220915	15/09/2022	1.0
PIS/CF	LSDDEP1_PIS_20220915	15/09/2022	1.0
PIS/CF	LSDDEP2_PIS_20220915	15/09/2022	1.0
PIS/CF	LSDDEP_Whanau_20220915	15/09/2022	1.0
Advertisement	Advertisement_Short	15/09/2022	1.0
Other	LSDDEP_DMC_Charter_v1	15/09/2022	1.0
Advertisement	LSDDEP1_LongAd	15/09/2022	1.0
Advertisement	LSDDEP2_LongAd	15/09/2022	1.0
Surveys/questionnaires	All questionnaires	15/09/2022	1.0
Response to PA Document	LSDDEP_Protocol_v2.0_tracked	01/11/2022	2.0
Response to PA Document	LSDDEP_PIS_GeneticAddendum	01/11/2022	2.0
Response to PA Document	LSDDEP_DataManagementPlan_tracked	01/11/2022	2.0
Response to PA Document	LSDDEP_DataManagementPlan_CLEAN	01/11/2022	2.0
Response to PA Document	LSDDEP1_PIS_tracked	01/11/2022	2.0
Response to PA Document	LSDDEP1_PIS_CLEAN	01/11/2022	2.0
Response to PA Document	LSDDEP2_PIS_tracked	01/11/2022	2.0
Response to PA Document	LSDDEP2_PIS_CLEAN	01/11/2022	2.0
Response to PA Document	CoverLetter	15/11/2022	1
Response to PA Document	Indemnity_Evidence	15/11/2022	1
Response to PA Document	Certificate_Clinical Trials_2022:2023_Newline	15/11/2022	1
Response to PA Document	LSDDEP_Protocol_v2.0_CLEAN	15/11/2022	2.0

Review Document Type	Review Document File Name	Review Document Version Date

Appendix B: Statement of compliance and list of members

Statement of compliance

The Southern Health and Disability Ethics Committee

- is constituted in accordance with its Terms of Reference
- operates in accordance with the *Standard Operating Procedures for Health and Disability Ethics Committees*, and with the principles of international good clinical practice (GCP)
- is approved by the Health Research Council of New Zealand's Ethics Committee for the purposes of section 25(1)(c) of the Health Research Council Act 1990
- is registered (number 00008713) with the US Department of Health and Human Services' Office for Human Research Protection (OHRP).

List of members

Mr Anthony Fallon (Lay (consumer/community perspectives)), Associate Professor Mira Harrison-Woolrych (Non-lay (intervention studies)), Dr Devonie Waaka (Non-lay (intervention studies)), Mr Dominic Fitchett (Lay (the law)), Ms Amy Henry (Non-lay (observational studies)), Associate Professor Nicola Swain (Non-lay (intervention studies)), Ms Dianne Glenn (Lay (consumer/community perspectives)), Ms Neta Tomokino (Lay (consumer/community perspectives)).

Unless members resign, vacate or are removed from their office, every member of HDEC shall continue in office until their successor comes into office (HDEC Terms of Reference).

http://www.ethics.health.govt.nz
